# Obstacles to the successful introduction of an electronic hand hygiene monitoring system, a cohort observational study

**DOI:** 10.1186/s13756-019-0498-2

**Published:** 2019-02-22

**Authors:** Phillip D. Levin, Reut Razon, Carmela Schwartz, Alexander Avidan, Charles L. Sprung, Allon E. Moses, Shmuel Benenson

**Affiliations:** 1 Unit for infection Prevention and Control, Department of Clinical Microbiology and Infectious Diseases, Hadassah-Hebrew University Medical Center, POB 12000, 9112001 Jerusalem, Israel; 2 Department of Anesthesiology, Hadassah-Hebrew University Medical Center, Jerusalem, Israel; 3 Hadassah-Hebrew University Medical Center, Jerusalem, Israel

**Keywords:** Hand hygiene, Surveillance, Electronic HH system

## Abstract

**Background:**

Hand hygiene (HH) compliance remains low in many intensive care units (ICU). Technology has been suggested to improve HH compliance.

We describe the introduction of an electronic HH surveillance and intervention system into the general ICU of a tertiary care teaching hospital, the obstacles to success and reasons for the system’s ultimate failure and removal.

**Methods:**

The system was based on radiofrequency transmitters in patient areas, on HH dispensers, and individual personal bracelets. The transmitters were connected to a central computer. The system was designed to detect entry and exit from patient areas and provide real time alerts of missed HH performance**.**

A staff satisfaction questionnaire was administered followed by validation of system accuracy. Electronic data were compared to human observer data collected during defined observation periods.

**Results:**

Data from 41 questionnaires revealed low satisfaction rate (21/41, 51%). Low system accuracy (31/41, 76%) and inconvenience (18/41, 44%) being the most frequent reasons.

During 44 one hour observation periods the observer recorded more HH opportunities and performances than the electronic system (mean number of HH opportunities/hour 10.9 ± 7.6 vs 6.8 ± 6.9, *p* < 0.001, correlation r = 0.75, p < 0.001, and performances/hour 8.7 ± 3.9 vs 6.0 ± 3.1, p < 0.001, correlation r = 0.60, p < 0.001, respectively). Correlation between observer and HH electronic system was very low (correlation coefficient r = 0.03, *p* = 0.91).

**Conclusions:**

The electronic HH system was not accepted by ICU staff principally due to inaccuracy and inconvenience. Inaccuracies were verified by direct observations. In order for an electronic HH system to succeed we suggest it must be highly accurate and comfortable to use.

## Background

Hand hygiene (HH) compliance remains appallingly low in intensive care units (ICUs) [1–4] and probably leads to the spread of bacteria which can be involved with subsequent hospital acquired infections [5–7].

Attempts to improve HH are generally based on five main elements: (1) improving access to HH facilities (2) training including educational messages delivered through lectures, (3) evaluation and feedback (4) posters etc. that are directed at a wide audience but are not specific to the individual, and (5) an improved culture of quality and safety [8, 9]. Fostering individual accountability or personal “ownership” of the HH issue is difficult using these tools. Possibly for these reasons, or due to the high intensity of HH

required during care of ICU patients (up to 11.4 ± 2.1 times per hour, or approximately every 5.5 min [10]), HH interventions are generally met with only limited or transient success [11].

Recently attempts have been made to exploit technology to personalize and thus improve HH compliance with variable success [12] [13]. A prerequisite for such systems is that they be accepted by the ICU staff, and that the data they provide are accurate.

We describe the process surrounding the introduction of an electronic HH surveillance and intervention system into the ICU. We present the results of a questionnaire relating to staff satisfaction regarding the system, and report a comparison of the incidence of HH opportunities, performance and compliance as recorded by the system and by an observer.

## Methods

In October 2013 an electronic HH surveillance and intervention system was introduced into the 13 bed General ICU of a tertiary academic medical center, for clinical use.

This report describes a questionnaire to identify causes of satisfaction/dissatisfaction with the electronic HH system, and a prospective assessment of the system’s accuracy. Both parts of the study were performed approximately three months after the introduction of the system. The hospital ethics committee approved performance of the study (0576–13-HMO) with agreement to cooperate being taken as consent to participate.

### Electronic hand hygiene system description

The electronic HH system was designed to identify entrance and exit from patient areas, regardless of whether there was contact with the patient [14]. This is the standard operating procedure in our ICU. The system was based on sensors connected to radio-frequency transmitters linked to a central computer. The sensors were widely distributed throughout the ICU, above every bed and on HH-product dispensers (both alcohol hand rub and 4% chlorhexidine gluconate soap dispensers). Each staff member was assigned a specific individual bracelet that was worn during working hours in the ICU. The bracelets were able to communicate with the sensors so that proximity to the patients’ beds (i.e. entrance and exit from an individual patient’s care area) and use of HH products could be monitored. All data were transmitted to the central computer. Real time information about whether HH was required or had been performed was then transmitted back to the staff members bracelet. If HH was required but had not been performed (defined as entrance and exit from a patient area), the bracelet vibrated and displayed a screen message. The system also measured adequacy of HH performance by measuring the time that hands were rubbed together – the bracelets included a motion sensor. Although greater than 15 s of hand rubbing was recommended, the system was designed to “accept” more than three seconds as adequate. In addition the system provided personal weekly reports (in the form of a cell phone text message to each staff member with their personal HH compliance percentage) and monthly HH compliance reports including specific data on all staff members sent to managers.

Prior to the introduction of the HH system in to the ICU, staff education sessions were performed on several occasions. The technical basis of the system was also described, and the importance of improving HH repeatedly presented. This led to considerable enthusiasm for using the system.

### Questionnaire

Staff satisfaction with the HH system was measured using a questionnaire. The questionnaire was delivered to physicians and nurses anonymously. The key question relating to satisfaction concerning the system was “To what extent are you satisfied with the electronic HH system?” 1 – not satisfied at all to 5 extremely satisfied. This question was followed by: “Please list three advantages associated with use of the system” and “Please list three disadvantages associated with use of the system”.

### Observer data

An observer was trained by an infection control nurse to identify HH opportunities and the use of HH products. A HH opportunity was defined as entrance or exit from an individual patient’s area – either the patient’s individual room or the area close to the patient’s bed including their personal equipment in the open plan area. Although the WHO five moments of HH includes other opportunities, the electronic system was designed to identify entrance and exit from patient areas only. Thus to ensure comparability, the observer was trained to identify similar opportunities. The length of time hands were rubbed together during use of HH products was also recorded by the observer. Adequacy of training was checked by the infection control nurse making parallel observation during an assessment period.

During each observation period a staff member (either a physician or a nurse) was asked to wear a “study bracelet” in addition to the clinical bracelet worn at all times. The study bracelet was used to collect electronic data for comparison with the observer data for the particular observation period only. The “clinical bracelet”continued to function as normal. Two dedicated “study bracelets” were used during the observation periods. Each observation period lasted one hour. A count of HH opportunities and HH performance was recorded for each observation period. The observer counts represented the gold standard for comparison to the same data provided by the electronic system for the same time periods.

### Data analysis

Due to technical limitations (known in advance), the electronic HH system was able to report only the total count of HH opportunities and the total count of HH performances for each individual one-hour observation period. These data did not describe whether HH had been performed in response to a specific opportunity and thus compliance could not be reported per individual one-hour observation period. The system was, however, able to provide compliance data for all observation periods performed during one week based on aggregation of multiple individual observation periods. This data was provided as a compliance rate per week only.

The mean number of HH opportunities and HH performance per one-hour observation period were compared for observer data and electronic data both graphically (Pearson correlation coefficient) and numerically (Students t-test). The weekly aggregate compliance rates were also compared. SAS 9.4 (Cary, NC, USA) was used for all statistical comparisons and significance was defined as two tailed *p* < 0.05.

## Results

A total of 41 questionnaires were filled out – 26 by nurses (out of 56, 46%) and 15 by doctors (out of 15, 100%). Satisfaction with the electronic HH system was low or very low for 21 of 41 staff members (51%). System inaccuracy (false positive notifications, or lack of notifications when required) represented the most common reason for dissatisfaction (31/41, 76%) followed by inconvenience of wearing the bracelet (18/41, 44%). The main advantages recorded were increased awareness and improved HH compliance (31/41, 76%), followed by organized uniform supervision for the entire staff (5/41, 12%).

**Fig. 1:**
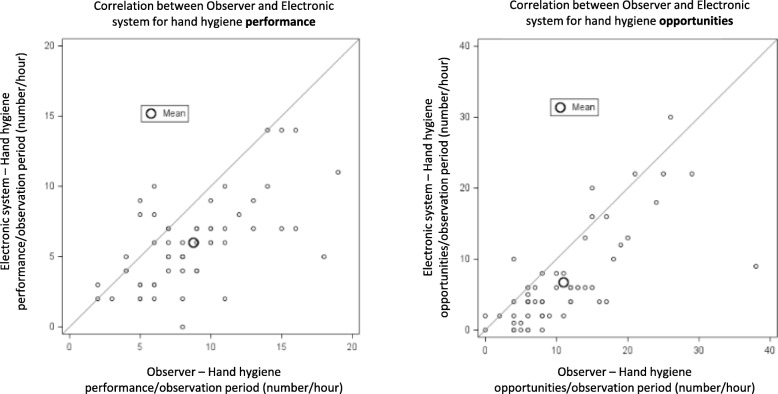
Correlation between observer and electronic system data for individual one-hour observation periods for the number of hand hygiene opportunities and performance

A total of 44 matched (observer and electronic) one-hour observation periods were analysed. During the observation periods a total of 613 HH opportunities and 487 HH performances were recorded by the observer vs 372 HH opportunities and 330 HH performances by the electronic system. The mean number of HH opportunities/hour was higher for the observer than the electronic system (10.9 ± 7.6 vs 6.8 ± 6.9, *p* < 0.001) as was the mean number of HH performance events/hour (8.7 ± 3.9 vs 6.0 ± 3.1, p < 0.001). Correlation between number of HH opportunities and performances per observation period for the observer and electronic system are shown graphically in Fig. 1 (Correlation coefficient: Observer vs electronic: number of HH opportunities/hour: r = 0.75, p < 0.001; number of HH performance events/hour: r = 0.60, p < 0.001).

**Fig. 2:**
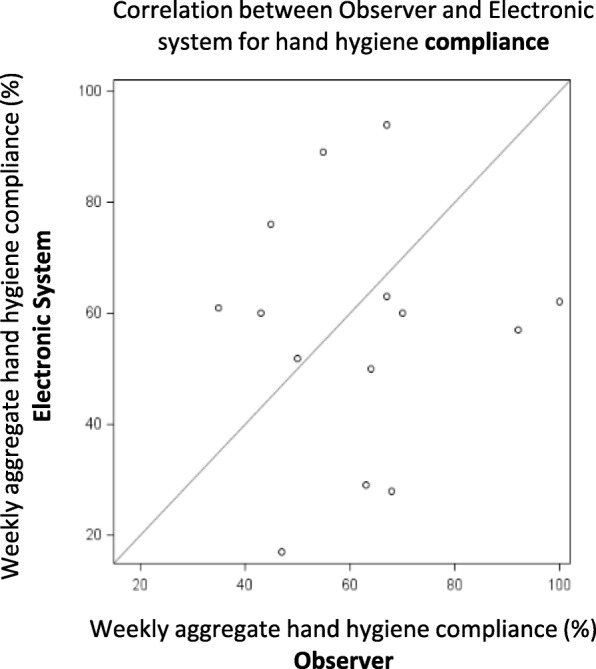
Correlation between observer and electronic system data for aggregate weekly hand hygiene compliance data

Due to a technical limitation of the electronic HH system, measured compliance data were available only weekly, rather than for each one-hour observation period. Weekly aggregation of data from the two study bracelets used yielded 14 weekly compliance data points. Correlation of mean weekly compliance between observer and HH electronic system is presented in Fig. 2. The correlation was found to be very low (correlation coefficient r = 0.03, *p* = 0.91).

## Discussion

Despite initial enthusiasm for the electronic HH system, this soon changed to disappointment and subsequently to unwillingness to continue with use of the system. The ICU staff appreciated the need for HH improvement (noted by 76% to be an important goal of system introduction), but equally were disappointed by the system’s poor performance (76%) and the inconvenience of wearing the required bracelet (44%). In terms of accuracy, staff dissatisfaction may have been justified, as the system consistently underestimated HH opportunities and HH performance when compared to direct observation and occasionally provided false alerts. Further, the weekly summary of HH compliance provided to staff was largely inaccurate. The number of HH opportunities per hour recorded by the observer in our study was similar to that reported elsewhere supporting the validity of the observer data [10].

The ability to monitor movements using technology seems attractive as a means of evaluating whether HH is performed properly and there are several commercial systems available today. These use radio-frequency transmission, cameras or and/or other sensors [15]. All these systems have been tested in simulated ICU environments but rarely in the real life situations – where the beds are close to one another, where there is little room between patient areas and where work load is high. For example Pineles et al. [16] studied the accuracy of “nGage” systems (a system based on radio-frequency and badges) and showed that the system had 88.5% accuracy in a simulated setting but during real-life clinical activities the system’s accuracy dropped to 50%.

Is imperfect accuracy a sufficient reason for not using an electronic HH system? The goal of using the system is not primarily to evaluate the staff performance accurately but rather to improve HH in order to prevent cross transmission of pathogens. It is possible that a relatively inaccurate system will still achieve this goal and benefit the patients. Two main obstacles stand before this approach however. First, in qualitative comments, the staff stated that if their performance was being scored, they felt they had the right to be scored accurately, and were disappointed when the system clearly “felt” inaccurate. Second, outside the ICU we are used to an extremely high level of technological performance – smartphones, tablets and laptops are extremely reliable, fast and accurate. The HH technology did not approach this level of function, and although it might have provided benefit, the frustration that resulted from imperfect function proved to be a major obstacle to acceptance.

It is interesting to note that the ICU staff was exquisitely sensitive to HH opportunities and performance. Within a short time they identified that the system was inaccurate giving false alarms and missing HH opportunities. Given that the staff seemed to be able to identify HH opportunities more accurately than the electronic system, one cannot avoid asking why overall HH compliance was only 65% as recorded by the observer.

Using the electronic HH system required staff members to wear a bracelet around their wrists. This is in contravention of the “bare below the elbows” approach, [17] which is mandated in the United Kingdom in order to ensure that the hands can be decontaminated throughout the duration of clinical work (in addition to removing wrist and hand jewellery etc.). This practice is based on biological plausibility, but the incremental infection prevention impact to inpatient care is unknown and mentioned as “may be considered” only in the guidelines published by the Society for Healthcare Epidemiology of America (SHEA) [18]. The bracelet used in our study, could be cleaned using antibacterial wipes, alcohol or even washed with antibacterial soap. Further, it is possible that improving HH is an objective of sufficient importance that may justify this infraction of the bare below the elbow practice.

Our research has several limitations. The electronic HH data represented the sum of data for one hour of observation periods and not single events. Therefore, we could not directly compare individual HH opportunities or events between the observer and the technology, but only numerical sums for each hour’s observation. Additionally, the system was unable to provide individual compliance data per one-hour observation period. However, compliance between observer and system could be compared based on available weekly aggregated data. We have chosen not to identify the specific technology used in this study as we did not directly measure whether the technology actually performed its task – to improve HH performance. Rather we looked at the reasons that the system ultimately failed and was removed from our ICU in order to suggest improvements required for acceptance.

## Conclusions

Staff dissatisfaction with the electronic system seemed to result primarily from inaccurate identification of HH opportunities and performance. The staff dissatisfaction seemed justified by direct observation of the electronic system. We suggest that for HH technology to be successfully introduced into the ICU it must be both highly accurate and comfortable to use.

## Abbreviations

HH: Hand hygiene
ICU: Intensive care unit
